# BGN/FAP/STAT3 positive feedback loop mediated mutual interaction between tumor cells and mesothelial cells contributes to peritoneal metastasis of gastric cancer

**DOI:** 10.7150/ijbs.72218

**Published:** 2023-01-01

**Authors:** Haitao Wu, Zhenxian Xiang, Guoquan Huang, Qiuming He, Jialing Song, Rongzhang Dou, Chaogang Yang, Shuyi Wang, Bin Xiong

**Affiliations:** 1 Department of Gastrointestinal Surgery, Zhongnan Hospital of Wuhan University, Wuhan, 430071, People's Republic of China.; 2 Department of Thyroid and Breast Surgery, Maternal and Child Health Hospital of Hubei Province, Tongji Medical College, Huazhong University of Science and Technology, Wuhan, 430071, People's Republic of China.; 3 Hubei Cancer Clinical Study Center, Wuhan, 430071, People's Republic of China.; 4 Hubei Key Laboratory of Tumor Biological Behaviors, Wuhan, 430071, People's Republic of China.; 5 Department of Gastrointestinal Surgery, Central Hospital of Enshi Tujia and Miao Autonomous Prefecture, Enshi, 445000, People's Republic of China.

**Keywords:** biglycan, cancer-associated fibroblasts, fibroblast activation protein, gastric cancer, peritoneal metastasis

## Abstract

Peritoneal metastasis (PM) is most frequent in gastric cancer (GC) and cancer-associated fibroblasts (CAFs) play a critical role in this process. However, the concrete mechanism of crosstalk between CAFs and cancer cells in PM of GC remains unclear. Microarray sequencing of GC focus and PM lesions was performed, and biglycan (BGN) was screened for further study. Clinically, BGN expression was higher in GC tissues than adjacent normal tissues, and high expression correlated with poor prognosis. *In vitro* experiments demonstrated that BGN promoted tumor progression and the transformation of mesothelial cells (MCs) into cancer-associated fibroblasts like cells (CAFLCs). In turn, CAFLCs-derived fibroblast activation protein (FAP) facilitated the proliferation, migration, invasion, and epithelial-mesenchymal transition (EMT) of GC cells. GC-derived BGN combined with toll like receptor 2 (TLR2)/TLR4 on MCs to activate the NF-κB pathway and promote the transformation of MCs into CAFLCs by the recovery experiment, coimmunoprecipitation assay, nuclear and cytoplasmic protein extraction assay. CAFLCs-derived FAP could activate the JAK2/STAT3 signaling pathway in GC. Finally, activated STAT3 promoted BGN transcription in GC, resulting in a BGN/FAP-STAT3 positive feedback loop. Taken together, mutual interaction between tumor cells and activated MCs mediated by a BGN/FAP-STAT3 positive feedback loop facilitates PM of GC and provides a potential biomarker and therapeutic target for GC metastasis.

## Introduction

Gastric cancer (GC) is the fifth most frequently diagnosed cancer and the third leading cause of cancer-related death worldwide 1. In advanced GC cases, 14% of patients have peritoneal metastasis (PM) during primary surgery, and the prognosis of patients with GC PM is extremely poor with a median survival time less than six months 2. Thus, there is an urgent need to explore the mechanism by which GC PM occurs to identify a poor prognostic factor which could contribute to PM of GC.

The metastatic niche in PM is primarily composed of abdominopelvic cavity surface, which is lined with a single layer of mesothelial cells (MCs). An intact peritoneum acts as the first line of defense against bacterial and tumor invasion 3. The interaction between tumor cells and human peritoneal MCs (HPMCs) promotes peritoneum secondary tumor implantation 4. Growing evidence displays that HPMCs are converted into CAFs through the mesothelial-to-mesenchymal transition (MMT), damaging the intact peritoneum and allowing tumor cells to colonize the peritoneum 5-8. CAF markers, which mainly consist of fibroblast activation protein (FAP), α-smooth muscle actin (α-SMA), and Vimentin, promote tumor progression 9-11. Studies also show that tumor cells exhibit more malignant behavior when co-cultured with CAFs 12-14.

Microarray sequencing is used to analyze differential expression of mRNAs between PM lesion and GC primary foci. Remarkably, according to microarray sequencing between PM foci and primary lesion, biglycan (BGN) ranked the fourth highest depending on fold change among 71 differential expression genes to screen for further studies. BGN, which is an extracellular protein (ECM) 15, is highly expressed in many human cancers, and predicts poor prognosis 16. In microarray and bioinformatic analysis data, BGN is associated with the epithelial-mesenchymal transition (EMT) process by integrating the TGFβ/Snail and TNFα/NF-κB pathway in the tumor microenvironment (TME) 17. In advanced bladder cancer, the expression of BGN correlates with the EMT process 18. EMT increases the ability of tumor epithelial cells to migrate and invade, enabling primary tumors to spread and form secondary tumor metastases 19, 20.

In the TME, Toll-like receptor 2 (TLR2) and Toll-like receptor 4 (TLR4) are considered the natural receptors of BGN, promoting tubular formation in endothelial cells by activating NF-κB signaling pathway 21. In colon cancer, BGN combines with TLR4 to activate NF-κB 22. Bioinformatics analysis of co-expression genes in BGN networks shows that BGN interacts with 42 neighboring genes, including FAP 16. While FAP is rarely detected in normal human tissue or benign tumors, this gene is up-regulated in stroma fibroblasts from more than 90% of malignant epithelial tumors 11. Studies demonstrate that FAP is one of the biomarkers for CAFs 9-11. However, it remains unknown whether BGN can combine with TLR2/TLR4 to activate NF-κB and promote the transformation of HPMCs into cancer-associated fibroblasts like cells (CAFLCs). In addition, the underlying mechanism by which CAFLCs impacts the PM of GC requires further research.

In this study, BGN expression in GC tissues was higher than paired adjacent normal tissues (PANT), and high expression correlated with poor prognosis of GC patients. *In vitro* experiments demonstrated that BGN promoted the GC progression and the transformation of MCs into CAFLCs. In turn, CAFLCs-derived FAP facilitated the proliferation, migration, invasion, and EMT of GC cells. FAP also rescued the effect of BGN on tumor progression. GC-derived BGN was shown to combine with TLR2/TLR4 on MCs to activate NF-κB and promote the transformation of MCs into CAFLCs. Moreover, CAFLCs-derived FAP activated the JAK2/STAT3 pathway in GC. Finally, STAT3 combined with the BGN promoter to induce BGN transcription, resulting in a BGN/FAP-STAT3 positive feedback loop in GC. This study shows that mutual interaction between tumor cells and activated MCs mediated by a BGN/FAP-STAT3 positive feedback loop facilitates PM of GC, providing a poor prognostic factor which could contribute to peritoneal metastasis of gastric cancer.

## Materials and Methods

### Microarray sequencing of GC focus and PM focus

Three pairs of tissue samples containing GC focus and PM focus were frozen and crushed using Biopulverizer, and the tissue samples were homogenized using Mini-Bead-Beater-16. Then Quick Amp Labeling, One-Color (Agilent p/n 5190-0442), RNeasy Mini Kit (Qiagen p/n 74104), and NanoDrop ND-1000 were utilized for labeling reaction, RNA purifying, and labeled cDNA QC respectively. After hybridization and microarray cleaning, scanning was performed using Agilent Microarray Scanner (Agilent p/n G2565BA). Finally, Agilent feature extraction software was used to extract all data.

### Patient tissue samples and follow-up

There were 65 sets of GC tissue and their PANT (more than 5 cm distance to cancer margin) samples in this study, all of which underwent radical surgery at Zhongnan Hospital of Wuhan University (Wuhan, China) from January 2016 to May 2018. Moreover, all of inclusion patients were diagnosed with primary GC by histopathology, and no patient received neo-adjuvant chemotherapy or local radiotherapy before radical operation. All of the severed tissue specimens were preserved in liquid nitrogen as soon as possible, and then stored at -80 °C. The last follow-up visit was June 10, 2021. TNM stages of GC were determined depending on the 8th edition of the AJCC Cancer Staging Manual. All of tissue samples were obtained with written informed from the patients, and the study was executed with the ethical approval for scientific research projects under medical ethics committee of Zhongnan Hospital of Wuhan University (Scientific Ethical Approval NO. 2019079).

### Cell lines, cell culture, and reagents

The human gastric cancer cell lines AGS, BGC823, HGC27, MGC803, MKN45, SGC7901, the human gastric epithelial cell line GES1, HPMCs line HMrSV5 were gained from the Cell Bank of Wuhan University in Wuhan. In the incubator, all cell lines were cultured in Dulbecco's modified Eagle's medium (DMEM, Gibco, USA) containing 10% fetal bovine serum (FBS, Gibco, USA) under a humidified atmosphere of 5% CO_2_ at 37 °C. To obtain CAFLCs, in the co-cultured model, the non-contact co-culture transwell system of six-well plate (Corning, USA) enabled the exchange of cell-secreted factors in the culture medium, while the penetration of cells themselves not allowed. 3×10^5^ HMrSV5 cells were seeded in the upper chamber, and 3×10^5^ cells GC cells or GES1 cells were seeded in the lower chamber of the co-cultured model in the DMEM containing 10% FBS for 12 hours (h) respectively. Then the cells were washed with phosphate-buffered saline (PBS, Gibco, USA) twice in the co-cultured model, and the culture medium was replaced with free-FBS DMEM until the morphological changes of HMrSV5.

Recombinant human FAP protein (rFAP) (ABclonal, China) was diluted with PBS and utilized at an ultimate concentration of 200 ng/ml. Stattic (STAT3 inhibitors), BAY 11-7082 (NF-κB inhibitors), C29 (TLR2 inhibitors), TAK242 (TLR4 inhibitors), PT100 (FAP inhibitors) was dissolved in Dimethyl sulfoxide (DMSO, Sinopharm, China), the above final concentrations were 10 μM, 5 μM, 50 μM, 50 μM, 100 μM separately. And our team purchased the above inhibitors from MedChemExpress, USA.

### Immunohistochemistry (IHC) staining

The expression of BGN and FAP in gastric tissue specimens including primary lesion, and PM lesion were detected by IHC. FAP, and Ki67 expression were measured *in vivo* xenograft assay by IHC. Specimens were treated with antibodies against human BGN (1:100, Novus Biologicals, USA), FAP (1:200, Bioswamp, China), Ki67 (1:200, Cell Signaling Technology, USA). IHC staining was conducted on paraffin-embedded formalin-fixed samples according to the instructions recommended by the manufacturer.

### Cell transfection

Lentiviral-mediated BGN overexpression and knockdown (Genechem, China) were constructed in GC cell lines. For knockdown of BGN, a total of three siRNAs and scrambled siRNA (JTS scientific, China) were transfected into GC cells with lipofectamine 2000 reagent (Invitrogen, USA) in Opti-MEM (Gibco, USA) for the sake of screening out the optimal knockdown effects. Then, according to the selected siRNA, lentivirus mediated knockdown of BGN vectors (anti-BGN) and their negative control lentivirus vectors (anti-NC-BGN) were constructed and transfected into GC cells. Moreover, lentivirus mediated overexpression of BGN vectors (Oe-BGN) and their negative control lentivirus vectors (Oe-NC-BGN) were also contracted and transfected into GC cells, the stable transfection cell lines expressed green fluorescent protein (GFP) and anti-puromycin. Following the manufacturer's protocol.

Two human short hairpin knockdown RNA of FAP (sh-FAP) plasmids and control plasmids (sh-control) were purchased from the Genechem, China.

### Quantification of secreting protein through enzyme-linked immunosorbent assay (ELISA)

The BGN and FAP concentrations in different culture supernatants were estimated by ELISA kits (Bioswamp, China) following the manufacturer's instructions.

### RNA isolation, reverse transcription, and quantitative real-time polymerase chain reaction (qRT-PCR)

The total RNA was isolated from GC cell lines, human tissue samples, primary GC and PM xenograft tumor cells by Trizol reagent (Invitrogen, USA) according to the instructions recommended by the manufacturer. The concentration of RNA was measured by Nanodrop 2000 spectrophotometer (Thermo Scientific, USA). Then, 1μg of total RNA from every specimen was reverse transcribed into cDNA by the PrimeScript^TM^ RT reagent kit (Vazyme, China). The SYBR-Green PCR Master Mix (Vazyme, China) and the cDNA were utilized for subsequent qRT-PCR by the BioRad IQ5 Real time PCR machine (BioRad, USA) to analysis mRNA expression. And Glyceraldehyde-3-phosphate dehydrogenase (GAPDH) was considered as an internal control. The 2-ΔΔCt method was used to calculate relative RNA expression. The sequences of primers involved in the assay were designed in Table S1 (supplementary Table 1).

### Western blot analysis

The total proteins in cells were washed with cold PBS twice and lysed in RIPA buffer containing protease inhibitor cocktail (Thermo Scientific, USA). Protein concentrations were quantified using the BCA Protein Assay Kit (Beyotime, China) after protein extraction. The whole proteins were separated by 10% SDS-PAGE and then electrotransferred onto PVDF membranes (Millipore, USA). After being blocked for 2h with 5% non-fat milk in tris-buffered saline including 0.1% tween-20 (TBST), the PVDF membranes were incubated with diluted primary antibodies in antibody dilatation buffer at 4 °C overnight. The following primary antibodies used were anti-BGN (1:1000, Novus Biologicals, USA), anti-TLR2 (1:1000, Proteintech, USA), anti-TLR4 (1:1000, Proteintech, USA), anti-FAP (1:1000, Bioswamp, China), anti-NF-κB (1:1000, Proteintech, USA), anti-p-NF-κB (1:1000, phosphor Ser536, Cell Signaling Technology, USA), anti-α-SMA (1:1000, Abcam, USA), anti-JAK2 (1:1000, Abcam, USA), anti-STAT3 (1:1000, Cell Signaling Technology, USA), anti-p-JAK2 (1:1000, phosphor Y1007+Y1008, Abcam, USA), anti-p-STAT3 (1:1000, phosphor Tyr705, Cell Signaling Technology, USA), anti-E-cadherin (1:1000, Proteintech, USA), Vimentin (1:1000, Proteintech, USA), anti-GAPDH (1:5000, Proteintech, USA), anti-PCNA (1:1000, Cell Signaling Technology, USA), anti-β-Actin (1:1000, Cell Signaling Technology, USA). The second day the PVDF membranes were washed fifth in TBST solution for 10 minutes each time, and subsequently incubated with diluted HRP-conjugated secondary antibodies for 1h at room temperature. Finally, the protein bands were scanned by the ChemiDoc^TM^ XRS+System (Bio-Rad, USA), and quantification was conducted using the Bio-Rad Image Lab software.

### Nuclear and cytoplasmic protein extraction assay

Nuclear and Cytoplasmic Protein Extraction Kit (Beyotime, Shanghai, China) was used to detect subcellular distribution of p65. Firstly, after co-culture with Oe-BGN AGS or Oe-NC-BGN AGS for 96h, HMrSV5 were used for subsequent experiments. The cells were fully resuspended in cytoplasmic protein extraction reagent A on ice for 15 minutes. Then cytoplasmic protein extraction reagent B was added and resuspended on ice for 1 minute. Next, the above cell suspension solution was centrifuged at 16000g for 5 minutes at 4 °C. The extracted supernatant was cytoplasmic protein. The sediment was fully resuspended using nuclear protein extraction reagent on ice for 30 minutes. Then, the above cell suspension solution was centrifuged at 16000g for 10 minutes at 4 °C. Finally, the extracted supernatant was nuclear protein.

### Coimmunoprecipitation (Co-IP) assay

Co-IP assay was used to analyze the binding relationship between the BGN and TLR2/TLR4 proteins. Firstly, HMrSV5 after being co-cultured with Oe-BGN AGS for 96h were used for subsequent experiments. We conducted Co-IP assay using the Co-IP Kit (Abison, Guangzhou, China) following the instructions recommended by the manufacturer. The cells were washed three times with cold PBS, then lysed in ice-cold lysis buffer on ice for at least 5 minutes. Next, the above cell suspension solution was centrifuged at 12000rpm for 10 minutes, and the supernatant was incubated with anti-BGN antibody, IgG, or TLR2 antibody, TLR4 antibody in shaking table for overnight at 4 °C. Next, the Protein A/G agarose beads were used to capture the antigen-antibody complexes for 12h at 4 °C, and then the beads were washed with PBS, and the complexes were boiled and subjected to western blot analysis.

### Dual-luciferase report assay

To assess whether rFAP regulate BGN expression via STAT3, A 2000-bp DNA fragment of the human BGN gene promoter containing STAT3 binding sites was amplified via PCR and cloned into pPRO-RB-Report-Basic plasmid (double report gene vector, RiboBio, China), including wild type (WT) of BGN promoter region plasmid, namely BGN-WT, mutation (MUT) of STAT3 potential binding BGN promoter region plasmid, namely BGN-MUT1 (-539 to -549), BGN-MUT2 (-1405 to -1415), BGN-MUT3 (-1468 to -1478), and BGN-negative control (BGN-NC) plasmid. Firstly, GC cells were seeded in six-well plates were transfected with BGN-NC or BGN-WT separately in culture solution containing stattic or DMSO for 24h, then the transfectants were suspended and replaced with fresh culture solution containing rFAP for 24h. Secondly, to further evaluate whether STAT3 regulate BGN expression directly, the human plasmid expression enhanced GFP STAT3 (STAT3-Ex) and corresponding negative control (STAT3-NC) were purchased from RiboBio, China. GC cells were seeded in six-well plates, and co-transfected with different plasmids containing BGN-WT, BGN-MUT1, BGN-MUT2, BGN-MUT3, BGN-NC, STAT3-Ex, STAT3-NC, then the transfectants were suspended and replaced with fresh culture solution. Finally, the total cells were lysed 48h after transfection, and relative luciferase activity were calculated by dual-luciferase reporter system (Progema, USA) following the instructions recommended by the manufacturer.

### Chromatin immunoprecipitation (CHIP) assay

To assess the interaction between STAT3 and BGN promoter region, we conducted CHIP assays using a SimpleCHIP Enzymatic Chromatin IP Kit (Cell Signaling Technology, #9003, USA) following the instructions recommended by the manufacturer. Firstly, the cells were fixed for protein/DNA crosslinking and lysed. Then the DNA was sheared using sonication and immunoprecipitated by anti-STAT3 antibody, and the normal rabbit immunoglobulin G (IgG) in the kit was used as a negative control. Subsequently, the unbound material was washed to purify bound antibody-protein/DNA material, then STAT3-bound DNA was released using proteinase K digestion and further purified by filtration. The purified DNA was analyzed by qRT-PCR via using the designed primers, and the primers contained the BGN promoter region spanning the sites of their corresponding binding with STAT3 (Table s1). Finally, the PCR products were analyzed on 2% agarose gel, and the gel containing ethidium bromide staining was visualized using Tanon-1600 Gel Image System (Tanon, China).

### Immunofluorescence staining (IF)

The cells were seeded on 15 mm round coverslip in 24-well plates and cultured for 24h. Then the cells were washed with PBS, fixed with 4% paraformaldehyde for 30 minutes, permeabilized with 0.1% Triton X-100 for 10 minutes and blocked in 3% bovine serum albumin for 20 minutes at room temperature. Sequentially, the cells were treated with primary antibodies including anti-FAP (1:100, Bioswamp, China), anti-Vimentin (1:100, Proteintech, USA), anti-WT1 (1:200, Cell Signaling Technology, USA), anti-mesothelin (1:200, Abcam, USA) at 4 °C overnight. The cells were rewashed with PBS and incubated with fluorescent secondary antibody for 50 minutes in the absence of light at room temperature. After being washed with PBS, the cell nuclei were stained with DAPI (10 μg/ml) for 10 minutes. Finally, the Olympus BX5 fluorescence microscope (Olympus Optical, Japan) was used to take the images of cells.

### Cell proliferation assay

Cell Counting Kit-8 (CCK8) and plate colony formation assay were performed to measure the cell proliferation. For CCK8 assay, the cells were seeded in 96-well plates with the cell density of 3.0×10^3^ cells/well in 100 μL culture solution. After incubating for 0h, 24h, 48h, 72h, 96h, each well was put in 10 μL CCK8 (Biosharp, China) and incubated for 2h at 37°C. Finally, the Multiskan FC microplate absorbance reader (Thermo Fisher, USA) was used to determine the OD values at a wavelength of 450 nm. For plate colony formation assay, 1.0×10^3^ viable cells were planted in 6-well plates and incubated for two weeks. Then the colonies were fixed with 4% paraformaldehyde for 30 minutes and stained with 0.5% crystal violet for 20 minutes. Finally, the colonies were photographed and recorded the number in 6-well plates.

### Wound healing assay

The wound healing assay was operated to detect the migratory ability of GC cells. The cells were planted in 6-well plates and incubated with the DMEM containing 10% FBS until they reached 90% confluence. Several vertical lines were scratched on the cell surface using 10 μL pipette tips, then the plates were washed with PBS to remove non-adherent cells and cells were cultured with the serum-free DMEM. Finally, the migrating cells photographs were captured after incubation for 0h, 24h. The area between both edges of cell free was detected using Image J software (NIH, USA).

### Migration assay and invasion assay

The migrated and invasive ability of tumor cells were estimated by Transwell migration and invasion assay 23-25. The migration assay and invasion assay were operated using a 24-well Transwells (8 μm pore size, Coring, USA). Cell migration assay was performed using 24-well Transwells uncoated with Matrigel, and Cell invasion assay was performed using 24-well Transwells pre-coated with Matrigel. Firstly, the upper chamber was precoated with 1:8 dilution of Matrigel (Corning, USA) in the Transwell invasion assay following the instructions recommended by the manufacturer, while the upper chamber was not treated with Matrigel in Transwell migration assay. For both assays, 1.0×10^5^ viable cells were seeded in upper chamber and cultured with serum-free DMEM, while 700 μL DMEM containing 10% FBS was added to the lower chambers. After 48h of incubation, the cells and Matrigel above the membrane in the upper chamber were carefully removed with cotton swabs. Then the migrated or invaded cells on the bottom surface of membrane were fixed with 4% paraformaldehyde and stained with 0.5% crystal violet. Finally, the stained cells in 5 random fields were counted and photographed under the inverted microscope at ×200 magnification to operate a quantitative analysis.

### *In vivo* xenograft assay

The 6-weeks old female BALB/c-nu mice were purchased from Gempharmatech (Jiangsu, China). All animal experiments were approved by laboratory animal welfare and ethics review of Wuhan University center for animal experiment (WP20210360). Firstly, the tumor growth assay was performed and randomly divided into five groups (n = 5 per group), namely anti-NC-BGN HGC27 (5.0×10^6^ cells) group, anti-BGN HGC27 (5.0×10^6^ cells) group, anti-BGN HGC27 (5.0×10^6^ cells) and CAFLCs/sh-control (5.0×10^6^ cells) group, anti-BGN HGC27 (5.0×10^6^ cells) and CAFLCs/sh-FAP (5.0×10^6^ cells) group, CAFLCs (5.0×10^6^ cells) group, which were resuspended in 200 μl of serum-free DMEM and Matrigel (Corning, USA) (1:1) and inoculated into the flank of each mice. For the tumor peritoneal dissemination assay, the number of cells in above groups was 1.0×10^7^ and also resuspended in 200 μl of serum-free DMEM and Matrigel (1:1), then injected into peritoneal cavity of each mouse. The mice in tumor growth assay and tumor peritoneal dissemination assay were sacrificed and necropsied to evaluate tumor burden after thirty days and forty days later respectively. Finally, the subcutaneous tumors and the intraperitoneal disseminated tumors were photographed and weighted to perform statistical analysis. Furthermore, the intraperitoneal disseminated tumors were detected by IHC staining, and qRT-PCR assay.

### Statistics analysis

All experiments were conducted at least in triplicate and results were presented as standard error of the mean (SEM). All statistical data were analyzed using Statistical Product and Service Solutions (SPSS) software version 22.0 (IBM SPSS, USA) and GraphPad prism software version 8.0 (GraphPad Software, USA) for windows. Pearson's correlation analysis was used to evaluate the correlation between mRNA expression of BGN and FAP in GC tissues. The relationship between the expression of BGN, FAP, and their pathological characteristics were analyzed using Chi-square test. Differences between the two groups were evaluated using the Student's *t* test. The Kaplan-Meier survival curve was drawn for survival analysis, and difference between curves was determined by the log rank test. Univariate and multivariate Cox regression analysis were performed to discriminate the independent factors of prognosis. A two-sided *P* < 0.05 was considered as statistically significant (*P < 0.05, **P < 0.01, ***P < 0.001).

## Results

### BGN expression is associated with poor overall survival (OS) in GC

**Figure 1:**
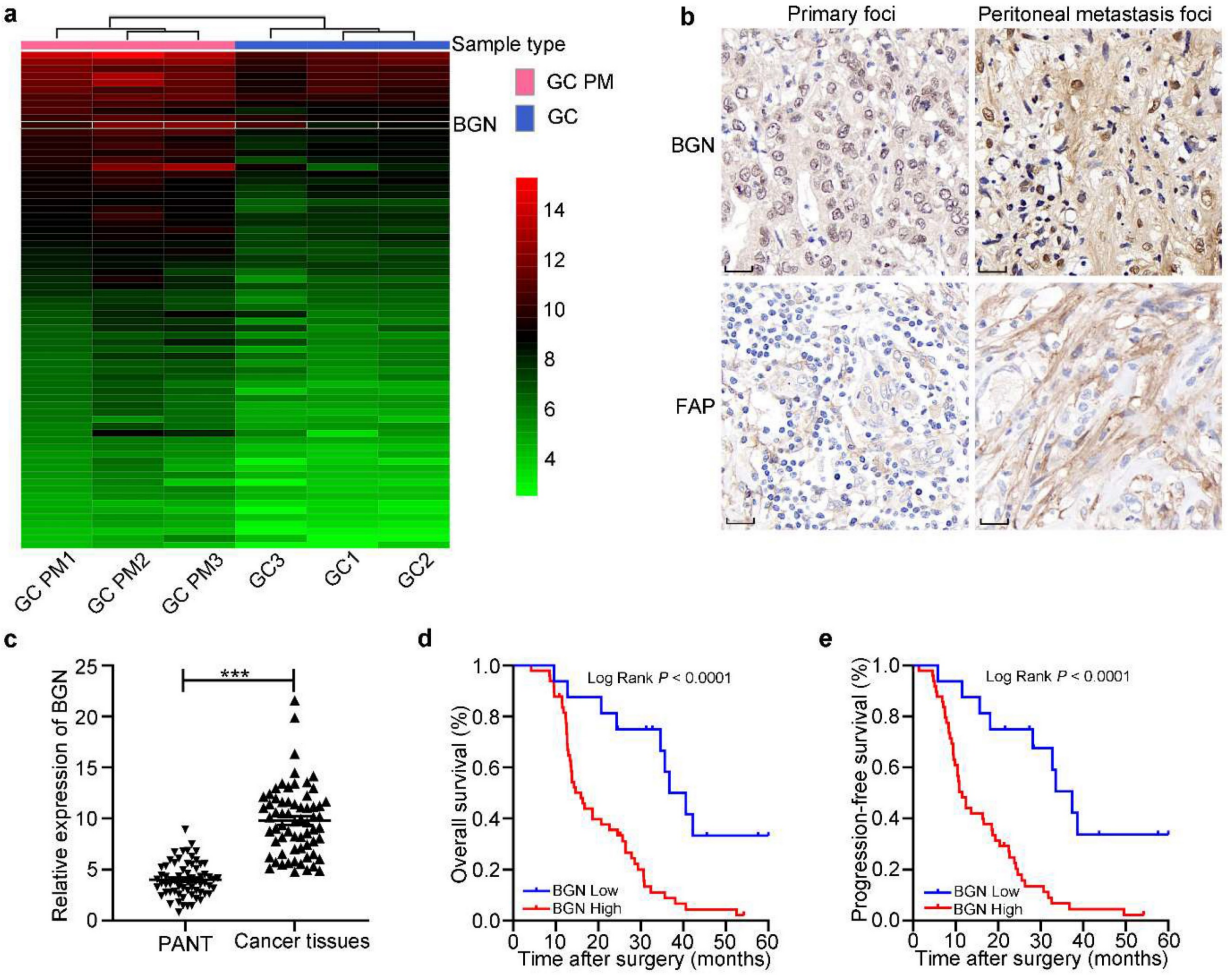
The expression and prognosis of BGN in gastric cancer. *P < 0.05, **P < 0.01, ***P < 0.001. a The heatmap of mRNA microarray sequencing between PM foci and GC primary lesion was analyzed in three pairs. Thus, BGN was screened for more study. **b** The representative IHC staining images for BGN and FAP expression were shown in GC primary foci and PM foci. Original magnification 400×, scale bar 20 μm. **c** The relative expression of BGN was measured by qRT-PCR in 65 GC tissues and paired adjacent normal tissues. **d**-**e** The association of BGN expression between five-year overall survival (d) and five-year progression-free survival (e) was analyzed by Kaplan-Meier survival analysis.

A heatmap of mRNA microarray sequencing between PM lesion and primary foci showed that the expression of BGN in PM of GC lesion was higher than that in primary GC lesion (Fig. 1a). BGN ranked the fourth highest depending on fold change among 71 differential expression genes to screen for further studies. IHC staining displayed that BGN expression was obviously higher in PM foci than GC primary foci, and was distributed in cytoplasm of tumor cells and stroma (Fig. 1b). In addition, qRT-PCR showed that BGN expression was higher in 65 GC tissues than matched PANT (Fig. 1c). To explore whether BGN expression was associated with GC progression, the correlation between BGN expression and clinicopathological parameters was assessed (Table 1). Importantly, high expression of BGN correlated significantly with T stage, LNM (lymph node metastasis), poor tumor differentiation degrees, advanced tumor-node-metastasis (TNM) stage, and postoperative PM (*P* < 0.05, respectively). Survival analysis demonstrated that patients with higher BGN expression had lower overall survival (OS) (*P* < 0.0001) (Fig. 1d) and progression-free survival (PFS) (*P* < 0.0001) (Fig. 1e). Univariate and multivariate analyses indicated that BGN expression was an independent prognostic factor correlating with poor PFS (HR = 4.733, 95%CI = 2.153-10.408, *P* = 0.000) and OS (HR = 4.851, 95%CI = 2.156-10.914, P = 0.000) (Table 2). These data showed that BGN facilitated tumor progression and influenced the prognosis of GC patients.

### BGN promotes the proliferation, migration, invasion, and EMT of GC cells

**Figure 2:**
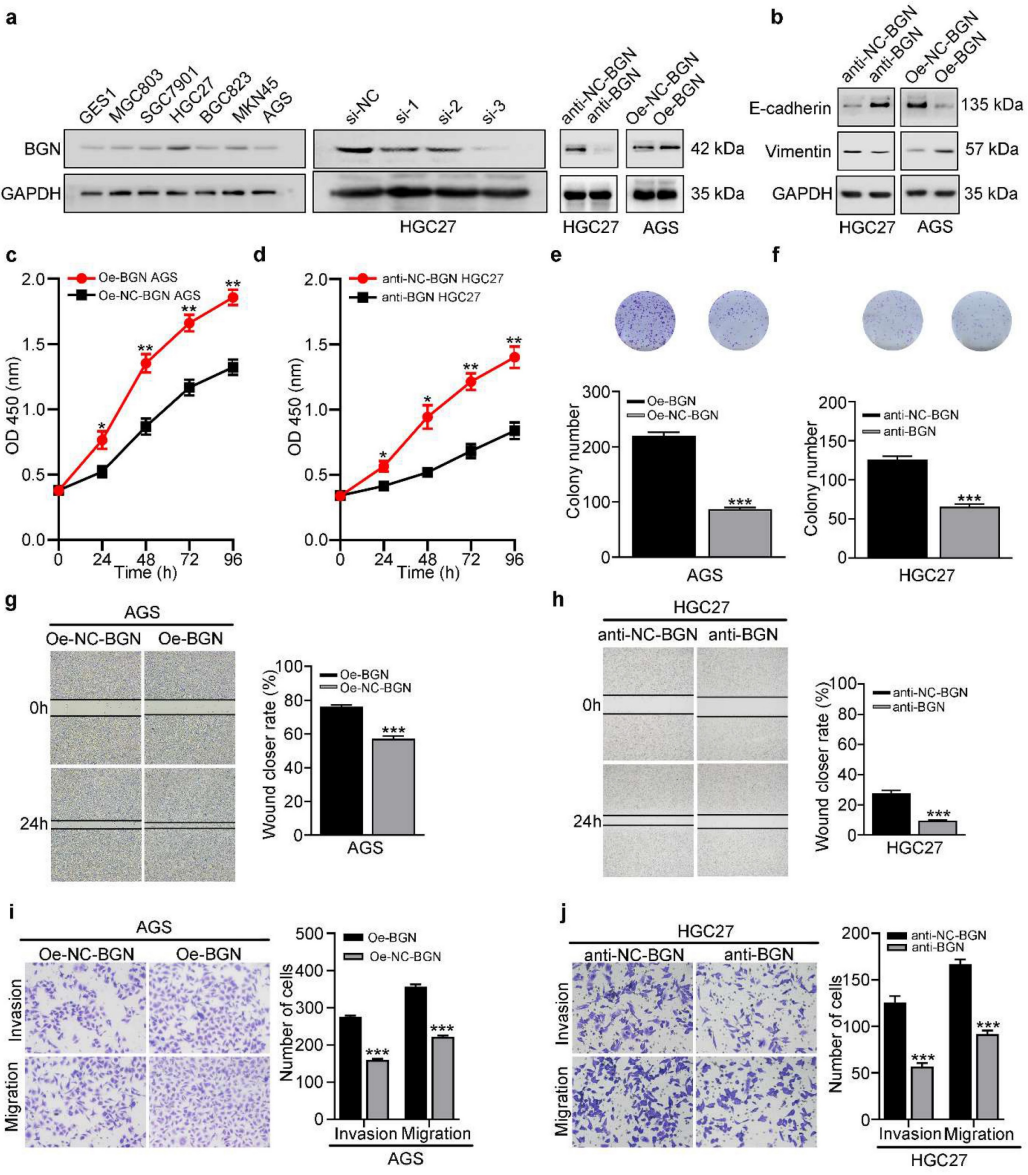
BGN promotes the proliferation, migration, invasion, and EMT of GC cells. *P < 0.05, **P < 0.01, ***P < 0.001. Data are shown as mean ± SEM (n = 3). a BGN expression was validated in six GC cell lines, the human gastric epithelial cell line (GES1). Three different siRNAs of BGN were designed and transfected into HGC27, and si-3 had the optimal knockdown effect. Lentiviral-based BGN knockdown stable HGC27 cell line (anti-BGN HGC27), lentiviral-based BGN overexpression stable AGS cell line (Oe-BGN AGS), and negative control stable cell lines were verified by western blot. **b-j** The effect of BGN on the proliferation, migration, invasion, and EMT of GC cells was detected with lentiviral-based BGN overexpression and knockdown stable cell lines by the CCK8 assay (c-d), colony formation assay (e-f), wound healing assay (g-h), Transwell migration and invasion assay (i-j), and western blot analysis (b), respectively. Representative photographs of wound healing (Original magnification 40×), migratory or invaded cells (Original magnification 200×).

To further explore the function of BGN, we firstly investigated the expression of BGN in GC cell lines. In contrast to GES-1, BGN was up-regulated in GC cell lines (Fig. 2a). While the maximum expression of BGN was detected in HGC27, the minimum expression was detected in AGS (Fig. 2a). Thus, these two cell lines were selected for additional experiments. Three different siRNAs were designed and transfected into HGC27, and si-3 had the optimal knockdown effect (Fig. 2a). Therefore, the si-3 sequence and corresponding negative control were selected to construct lentivirus-based vectors. Lentivirus-mediated BGN overexpression was constructed in AGS. Then the down-regulation and up-regulation expression of BGN effects were detected by western blot (Fig. 2a). Gain and loss of function experiments demonstrated that overexpression of BGN in AGS promoted the proliferation (Fig. 2c, 2e), migration (Fig. 2g, 2i), and invasion (Fig. 2i) of GC cells, while knockdown of BGN in HGC27 inhibited the proliferation (Fig. 2d, 2f), migration (Fig. 2h, 2j), and invasion (Fig. 2j) of GC cells.

In the context of neoplasia, EMT is closely linked with migration and invasion of cancer cells and plays an important role in cancer metastasis. To assess whether EMT is implicated in BGN-induced migration and invasion, western blot was used to measure expression of the epithelia marker (E-cadherin), and the mesenchymal marker (Vimentin). Overexpression of BGN in AGS significantly facilitated Vimentin expression and decreased E-cadherin expression (Fig. 2b), while knockdown of BGN in HGC27 dramatically inhibited Vimentin expression but improved E-cadherin expression (Fig. 2b). Taken together, these results indicate that BGN can promote the proliferation, migration, invasion, and EMT of GC cells.

### BGN promotes the transformation of HMrSV5 into CAFLCs through TLR2/TLR4/NF-κB signaling pathway

**Figure 3:**
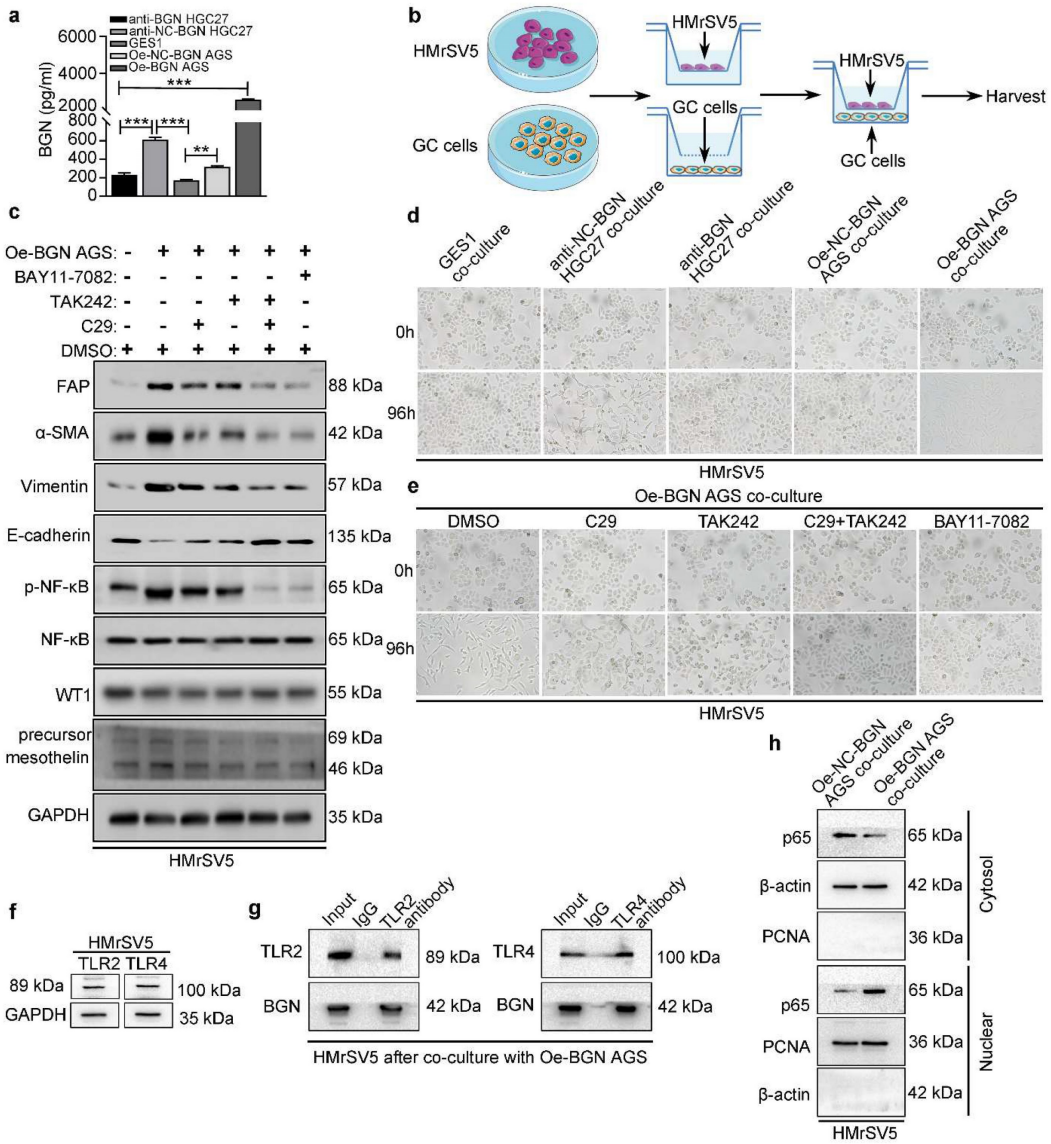
In co-cultured model, BGN promotes the transformation of HMrSV5 into CAFLCs through TLR2/TLR4/NF-κB signaling pathway. *P < 0.05, **P < 0.01, ***P < 0.001. Data are shown as mean ± SEM (n = 3). a ELISA assay of BGN protein secretion of GES1, anti-BGN HGC27, anti-NC-BGN HGC27, Oe-BGN AGS, and Oe-NC-BGN AGS. **b** Schema for representing the experiment procedures. **c** and** e** The effect of Oe-BGN AGS on the transformation of HMrSV5 was analyzed by western blot (c) and bright-field images (e). HMrSV5 were incubated with DMSO, C29, TAK242, C29+TAK242, or BAY11-7082 for 24h prior to co-culture with Oe-BGN AGS for 96h. Representative bright-field photographs (Original magnification 200×). **d** The effect of GES1, anti-BGN HGC27, anti-NC-BGN HGC27, Oe-BGN AGS, or Oe-NC-BGN AGS on the transformation of HMrSV5 for 96h was analyzed by bright-field images. Representative bright-field photographs (Original magnification 200×). **f** TLR2 and TLR4 expression were validated in HMrSV5 by western blot. **g** Coimmunoprecipitation (Co-IP) assays were used to identify interaction between BGN and TLR2/TLR4 in HMrSV5 after co-culture with Oe-BGN AGS for 96h. **h** Nuclear and cytoplasmic protein extraction assays were evaluated by western blot in HMrSV5 after co-culture with Oe-BGN AGS or Oe-NC-BGN AGS for 96h.

**Figure 4:**
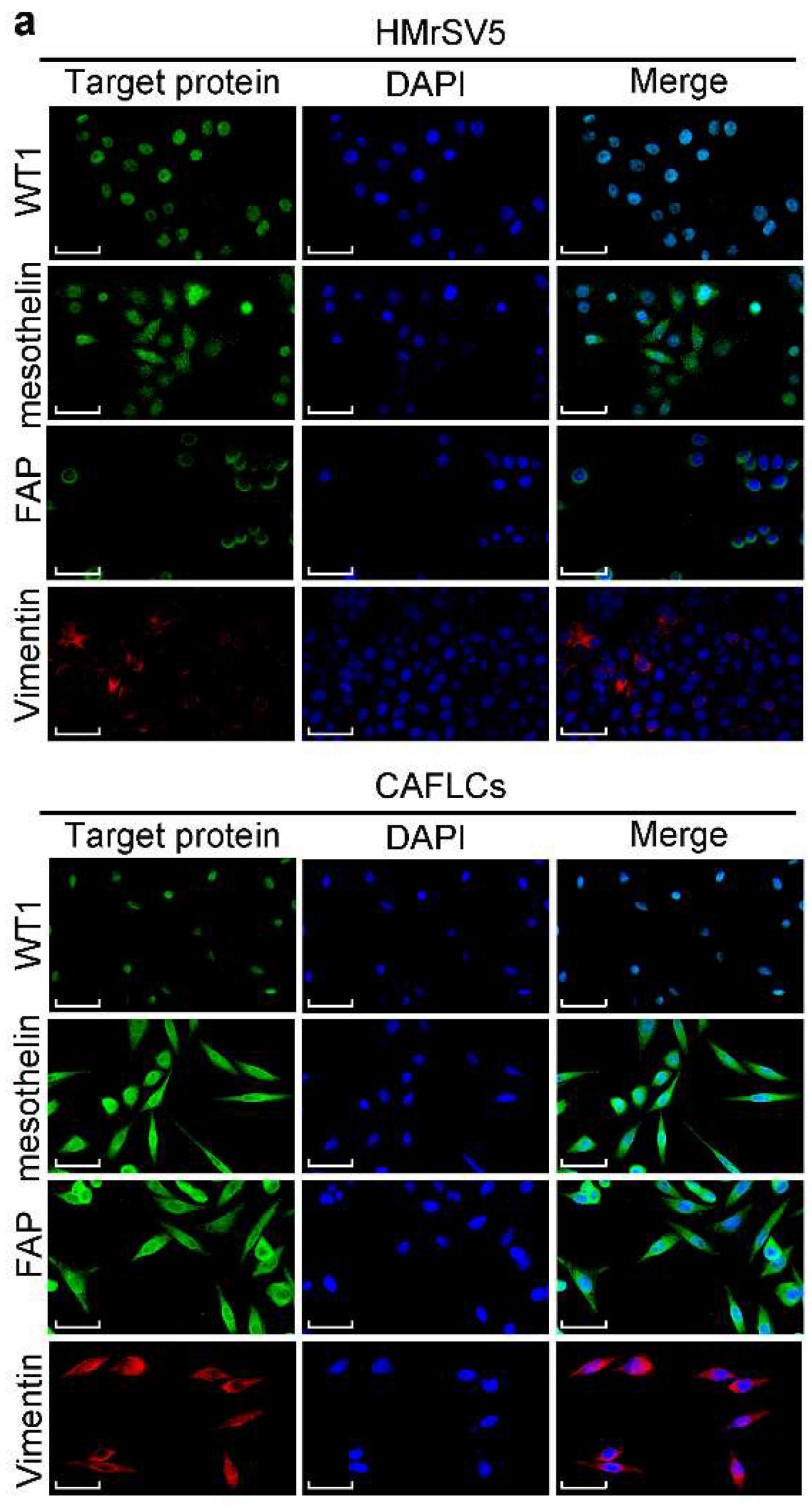
** The expression of related proteins between CAFLCs and HMrSV5 are detected by Immunofluorescence assays. a** Immunofluorescence assays of WT1, mesothelin, FAP, and Vimentin in CAFLCs and HMrSV5 were detected respectively. Original magnification 400×, scale bar 50 μm.

To further assess the function of BGN in TME, the concentration of BGN in culture medium of GES1, anti-BGN HGC27, anti-NC-BGN HGC27, Oe-BGN AGS, and Oe-NC-BGN AGS, was measured by ELISA. BGN levels were highest in supernatant of Oe-BGN AGS (Fig. 3a). Then we took advantage of co-culture experiment (Fig. 3b). Each of the cell lines listed above was co-cultured with HMrSV5 respectively. After co-culture with HMrSV5 for 96h, we observed that the normal cobble-stone morphology of HMrSV5 transmuted into spindle-like morphology in the co-culture group of Oe-BGN AGS, and partial changes occurred in the anti-NC-BGN HGC27 group (Fig. 3d). Vimentin, α-SMA, and FAP expression were significantly up-regulated in HMrSV5 after co-culture with Oe-BGN AGS, while E-cadherin expression was down-regulated compared with HMrSV5 after incubation with DMSO (Fig. 3c). Similar findings were observed in IF assay (Fig. 4a). Moreover, WT1 and mesothelin were two main markers of mesothelial cells, Western blot and IF assays showed that both HMrSV5 and HMrSV5 after co-culture with Oe-BGN AGS expressed WT1 and mesothelin (Fig. 3c, Fig. 4a). In summary, these data show that GC cells-derived BGN can convert HMrSV5 into CAFLCs by MMT in TME.

BGN is the ligand for TLR2/TLR4, and NF-κB is an extremely important downstream signaling pathway of TLR2/TLR4. However, whether GC cells-derived BGN can combine with TLR2/TLR4 receptors to transform HMrSV5 into CAFLCs remains to be explored. Expression of TLR2/TLR4 in HMrSV5 was detected at the protein level (Fig. 3f). Next, HMrSV5 were incubated with DMSO, C29 (TLR2 inhibitor), TAK242 (TLR4 inhibitor), C29+TAK242, or BAY11-7082 (NF-κB inhibitor) for 24h prior to co-culture with Oe-BGN AGS. Compared with DMSO, C29+TAK242 or BAY11-7082 fully blocked the transformation of HMrSV5 into CAFLCs, while C29 or TAK242 partially blocked the transformation by the recovery experiment (Fig. 3c, 3e). Moreover, the Co-IP results indicated that BGN interacted with TLR2 and TLR4 in HMrSV5 after co-culture with Oe-BGN AGS (Fig. 3g). Meanwhile, Nuclear and cytoplasmic protein extraction assays were used to detect cytosolic and nuclear p65 distribution in HMrSV5 after co-culture with Oe-BGN AGS or Oe-NC-BGN AGS. The results indicated that p65 nuclear translocation markedly increased in HMrSV5 after being co-cultured with Oe-BGN AGS compared with HMrSV5 after being co-cultured with Oe-NC-BGN AGS (Fig. 3h). Therefore, Oe-BGN AGS-derived BGN promoted p65 nuclear translocation of HMrSV5 in co-culture model. Taken together, these results show that GC cells-derived BGN can convert HMrSV5 into CAFLCs through TLR2/TLR4/NF-κB signaling pathway in TME.

### CAFLCs-derived FAP promotes tumor progression in TME

**Figure 5:**
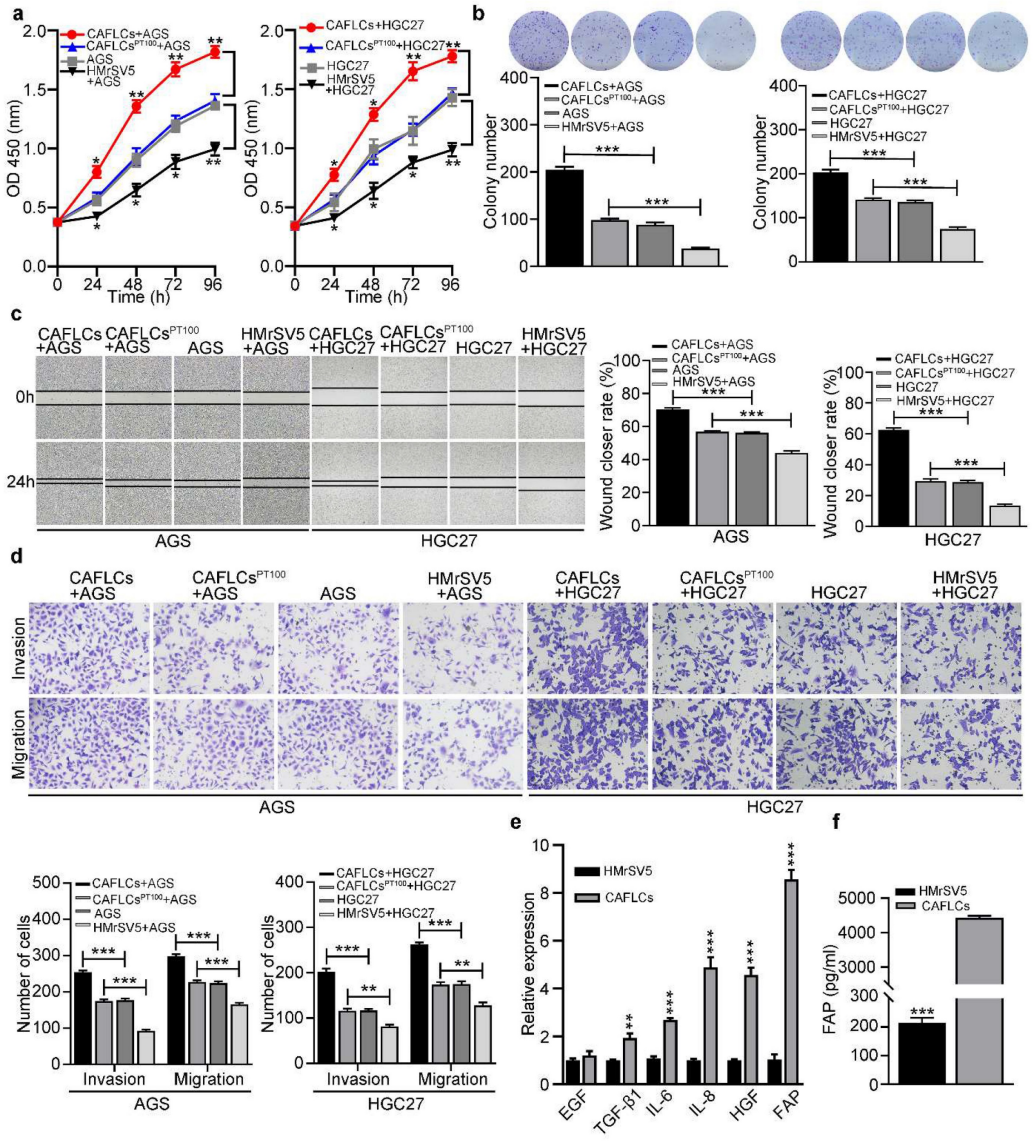
In co-cultured model, CAFLCs-derived FAP facilitates the proliferation, migration, and invasion of GC cells. *P < 0.05, **P < 0.01, ***P < 0.001. Data are shown as mean ± SEM (n = 3). a-d The effect of HMrSV5 or CAFLCs-derived FAP on the proliferation, migration and invasion of GC cells (AGS or HGC27) was detected by the CCK8 assay (a), colony formation assay (b), wound healing assay (c), Transwell migration and invasion assay (d), respectively. HGC27 or AGS were separately co-cultured with CAFLCs, HMrSV5, or CALCFs^PT100^ for 24h. Representative photographs of wound healing (Original magnification 40×), migratory or invaded cells (Original magnification 200×). **e** Relative expression levels of representative CAFLCs-secreted key cytokines were measured by qRT-PCR.** f** ELISA assay of FAP protein secretion of CAFLCs and HMrSV5.

**Figure 6:**
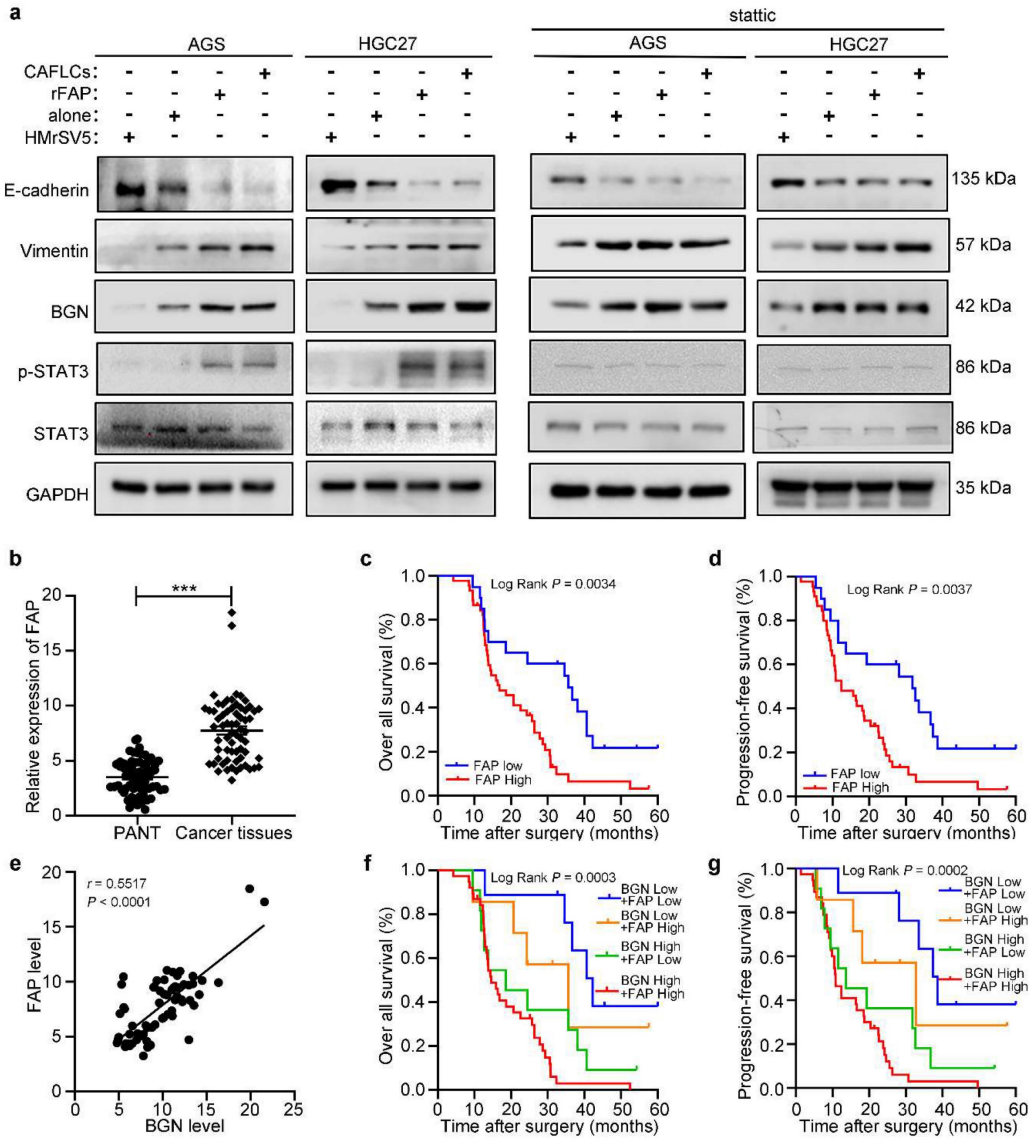
The expression and prognosis of FAP in gastric cancer. *P < 0.05, **P < 0.01, ***P < 0.001. Data are shown as mean ± SEM (n = 3). a Western blot analysis of GC cells (AGS or HGC27) alone, rFAP-supplemented GC cells, HMrSV5-co-cultured GC cells, CAFLCs-co-cultured GC cells in the presence or absence of stattic. **b** The relative expression of FAP was detected by qRT-PCR in 65 GC tissues and paired adjacent normal tissues. **c**-**d** The association of FAP expression between five-year overall survival (c) and five-year progression-free survival (d) was analyzed by Kaplan-Meier survival analysis. **e** The correlation of BGN expression with FAP expression in GC tissues. **f-g** The association of both BGN and FAP expression between five-year overall survival (f) and five-year progression-free survival (g) were analyzed by Kaplan-Meier survival analysis.

To further investigate the role of HMrSV5-derived CAFLCs in TME, HGC27 or AGS were separately co-cultured with CAFLCs or HMrSV5 for 24h. While CAFLCs increased the proliferation (Fig. 5a, 5b), migration (Fig. 5c, 5d), invasion (Fig. 5d), and EMT (Fig. 6a) of GC cells, HMrSV5 inhibited these effects. Next, the mechanism by which CAFLCs promote tumor progression was assessed. Given the important role of CAFs derived-cytokines in tumorigenesis and development, changes in the expression of six key cytokines in CAFs were assessed 9, 10, 26, 27. In contrast to HMrSV5, FAP was most up-regulated in CAFLCs (Fig. 5e). ELISA results also showed that FAP was more abundant in supernatant of CAFLCs than HMrSV5 supernatants (Fig. 5f). To evaluate whether CAFLCs-derived FAP had pro-tumorigenic activities *in vitro*, HGC27 or AGS were co-cultured with CAFLCs or CAFLCs^PT100^ (CAFs pretreated with PT100) for 24h. The results showed that CAFLCs derived-FAP evidently promoted proliferation (Fig. 5a, 5b), migration (Fig. 5c, 5d), invasion (Figure 5d), and EMT (Fig. 6a) of GC cells.

Clinically, IHC staining was performed to measure FAP expression in primary GC foci and PM foci from patient, and found to be markedly higher in PM foci than GC primary foci (Fig. 1b). Then FAP expression in 65 GC tissues was higher than matched PANT via qRT-PCR (Fig. 6b). FAP expression had similar results with BGN in GC, including clinicopathological parameters (Table 1), OS (*P* = 0.0034) (Fig. 6c), PFS (*P* = 0.0037) (Fig. 6d), and univariate analyses (Table 2). Moreover, we showed that BGN was positively correlated with FAP expression by qRT-PCR in GC tissues (Fig. 6e), and patients with high expression of both BGN and FAP had the worst survival outcome (Fig. 6f, 6g).

### FAP promotes BGN expression in GC cells by activating STAT3, and creating a positive feedback loop

**Figure 7:**
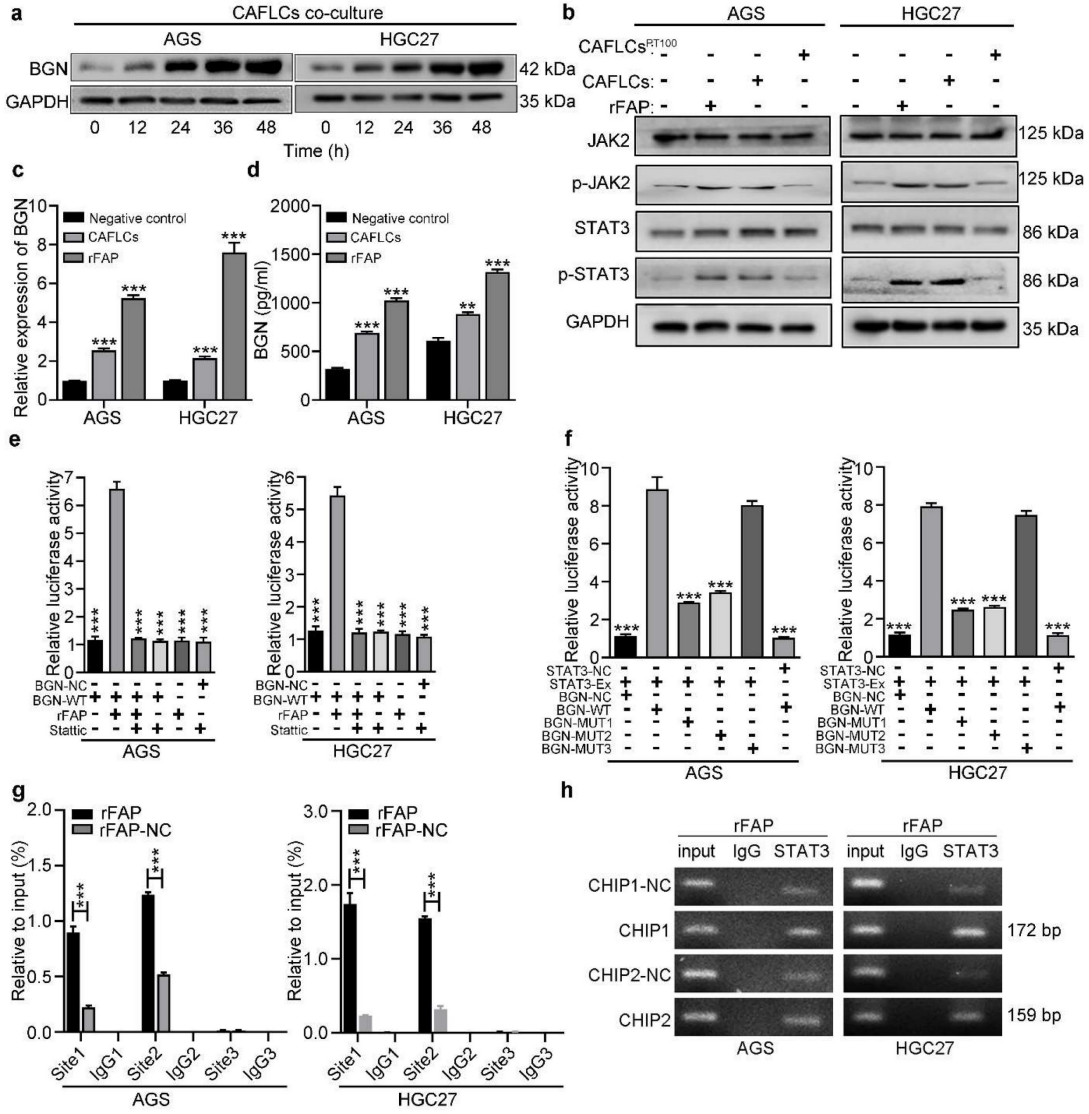
FAP promotes BGN expression in GC cells by activating STAT3. *P < 0.05, **P < 0.01, ***P < 0.001. Data are shown as mean ± SEM (n = 3). a Western blot of BGN from GC cells (AGS or HGC27) co-cultured with CAFLCs in a time-dependent manner. **b** Western blot analysis of GC cells (AGS or HGC27) alone, rFAP-supplemented GC cells, CAFLCs-co-cultured GC cells, and CAFLCs^PT100^-co-cultured GC cells for 48h. **c** The relative expression of BGN was detected in GC cells (AGS or HGC27) alone, rFAP-supplemented GC cells, CAFLCs-co-cultured GC cells for 48h**. d** The expression of BGN was detected in GC cells (AGS or HGC27) alone, rFAP-supplemented GC cells, CAFLCs-co-cultured GC cells for 48h by ELISA assay. **e** GC cells (AGS or HGC27) were transfected with a BGN promoter reporter plasmid (pPRO-RB-Report-Basic plasmid) for 24h**.** GC cells were incubated with or without stattic (10 μM) for 24h before rFAP (200 ng/ml) stimulation, relative luciferase activity of BGN promoter was detected after rFAP stimulation for 24h. **f** A reporter plasmid for BGN was constructed by cloning BGN promoter region (WT or NC) or identified STAT3 binding site mutants (MUT-1, MUT-2, MUT-3), and the human plasmid expression STAT3 (STAT3-Ex, STAT3-NC) were co-transfected with GC cells (AGS or HGC27), relative luciferase activity of BGN promoter was detected 48h after transfection. **g-h** GC cells (AGS and HGC27) were stimulated with rFAP. The qRT-PCR of CHIP products demonstrated the direct binding ability of STAT3 to BGN promoter region in GC cells, the input (2%) (g). The qRT-PCR of CHIP products was analyzed including negative control (NC), CHIP1, CHIP2, and CHIP3. The values were normalized to input (2%), and agarose gel (2%) (h).

Findings indicated that BGN and FAP expression were positively correlated, and bioinformatics analysis of co-expression genes showed that BGN interacted directly with FAP 16. Interestingly, CAFLCs could promote BGN expression in GC cells in a time-dependent manner (Fig. 7a). These findings inspired us to explore whether CAFLCs derived-FAP could regulate BGN expression. HGC27 or AGS were separately incubated with rFAP or co-cultured with CAFLCs for 48h. The results displayed that rFAP or CAFLCs could promote the expression of BGN in GC cells (Fig. 6a, 7c, 7d). As previously reported, JAK2/STAT3 signaling pathway could be activated by CAFs or FAP stimulation 9, 28-30. Thus, it was speculated that CAFLCs-derived FAP could promote BGN expression in GC cells through JAK2/STAT3 signaling pathway. HGC27 or AGS were separately co-cultured with CAFLCs, CAFLCs^PT100^, or incubated with rFAP for 48h. The results showed that p-JAK2 and p-STAT3 expression in GC cells significantly increased after co-culture with CAFLCs or incubation with rFAP but markedly decreased after co-culture with CAFLCs^PT100^ (Fig. 7b). Thus, CAFLCs-derived FAP played a vital role in GC cells by activating JAK2/STAT3 signaling pathway. Next, HGC27 or AGS were separately treated with stattic (a STAT3 inhibitor) for 24h prior to co-culture with CAFLCs, HMrSV5, or rFAP. The results displayed that rFAP or CAFLCs could promote BGN and p-STAT3 expression, and EMT of GC cells, while stattic significantly inhibited these effects (Fig. 6a). Taken together, these results suggest that CAFLCs-derived FAP boosted BGN expression and EMT of GC cells by activating JAK2/STAT3 signaling pathway.

A dual-luciferase assay was performed to further explore whether FAP could directly activate the BGN promoter region by STAT3 signaling pathway. HGC27 or AGS were transfected with dual-luciferase vector of BGN (WT or NC), and the transfected cells were incubated with or without stattic before rFAP simulation. FAP was shown to promote BGN transcription in GC cells by activating STAT3 (Fig. 7e). Bioinformatics prediction was conducted using Jaspar to uncover potential transcription binding sites on the BGN promoter region and find three putative STAT3-binding sites. A dual-luciferase vector of BGN-MUT that included BGN-MUT1 (-539 to -549), BGN-MUT2 (-1405 to -1415), and BGN-MUT3 (-1468 to -1478), and a plasmid of STAT3-Ex and STAT3-NC were constructed. To detect involvement of STAT3 in BGN regulation, every BGN report, including BGN-NC, BGN-WT, BGN-MUT1, BGN-MUT2, and BGN-MUT3, were co-transfected with STAT3-Ex or STAT3-NC in HGC27 or AGS, and promoter activity was assessed 48h after transfection. The result showed that BGN-MUT1 and BGN-MUT2 obviously decreased STAT3-Ex activation of the BGN promoter in contrast to the activation observed in response to BGN-WT or BGN-MUT3 (Fig. 7f). Findings from the dual-luciferase assay implied that BGN-MUT1 (-539 to -549) or BGN-MUT2 (-1405 to -1415) might contain a STAT3-binding site. Three primer sets were designed for potential STAT3 binding sites, site1, site2, and site3 (table S1) using a CHIP assay. Findings indicated that STAT3 bound directly to two STAT3 binding sites, site1 and site2 (Fig. 7g, 7h). These results demonstrated that CAFLCs-derived FAP could promote BGN transcription by activating STAT3 in GC cells to form a positive feedback loop.

### GC cells-derived BGN and CAFLCs-derived FAP promote GC PM *in vivo*

**Figure 8:**
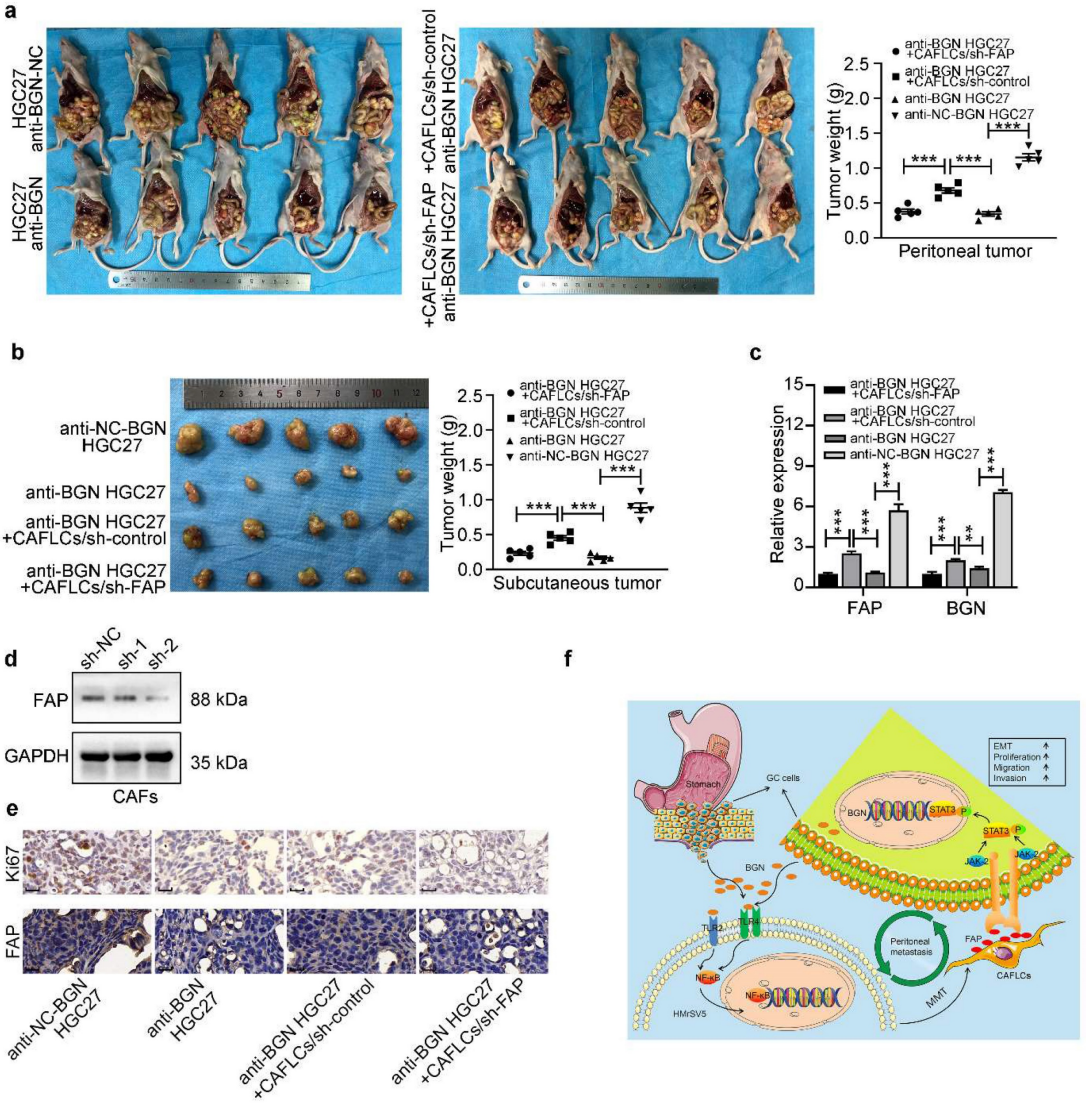
Co-injection of GC cells-derived BGN and CAFLCs-derived FAP promote GC PM *in vivo*. *P < 0.05, **P < 0.01, ***P < 0.001. Data are shown as mean ± SEM (n = 3). a-b The morphological characteristics of peritoneal and subcutaneous tumor xenograft, tumor weight in anti-NC-BGN HGC27 group, anti-BGN HGC27 group, anti-BGN HGC27+CAFLCs/sh-control group, anti-BGN HGC27+CAFLCs/sh-FAP group. Each group had five mice. **c** The relative expression of BGN and FAP mRNA of peritoneal tumor from anti-NC-BGN HGC27 group, anti-BGN HGC27 group, anti-BGN HGC27+CAFLCs/sh-control group, anti-BGN HGC27+CAFLCs/sh-FAP group. **d** Two different shRNAs of FAP were designed and transfected into CAFLCs, and FAP (sh-2) obtained a better knockdown effect by western blot. **e** IHC analyzed Ki67 and FAP expression in peritoneal tumor from anti-NC-BGN HGC27 group, anti-BGN HGC27 group, anti-BGN HGC27+CAFLCs/sh-control group, anti-BGN HGC27+CAFLCs/sh-FAP group. Original magnification 400×, scale bar 20 μm.** f** Schematic illustration of mutual interaction between tumor cells and activated MCs in the tumor microenvironment. Our study proposed the mechanism model that a BGN/FAP-STAT3 positive feedback loop facilitated PM of GC.

To confirm the* in vitro* results, an *in vivo* xenograft gastric cancer nude mouse model was performed. Anti-NC-BGN HGC27, anti-BGN HGC27, anti-BGN HGC27+CAFLCs/sh-control, anti-BGN HGC27+CAFLCs/sh-FAP, and CAFLCs groups were injected subcutaneously or intraperitoneally into nude mice. Plasmid was transfected to knockdown expression of FAP into CAFLCs. As shown Fig. 8d, FAP (sh-2) obtained a better knockdown effect. Thus, the second plasmid (sh2-FAP) was selected for the experiment. No tumors were formed after subcutaneous or intraperitoneal injection of CAFLCs alone (data not shown).

For the subcutaneous xenograft model (Fig. 8b), the weight of tumors in anti-NC-BGN HGC27 group were noticeably heavier than those in anti-BGN HGC27 group, anti-BGN HGC27+CAFLCs/sh-control group, anti-BGN HGC27+CAFLCs/sh-FAP group, further analysis showed the weight of tumors in anti-BGN HGC27+CAFLCs/sh-control group were obviously heavier than those in anti-BGN HGC27 group, anti-BGN HGC27+CAFLCs/sh-FAP group, while two groups of anti-BGN HGC27, anti-BGN HGC27+CAFLCs/sh-FAP were not statistically significant in tumor weights.

For the tumor peritoneal dissemination assay (Fig. 8a), the tumors produced by anti-NC-BGN HGC27 were dramatically heavier and wider than those anti-BGN HGC27, anti-BGN HGC27+CAFLCs/sh-control, anti-BGN HGC27+CAFLCs/sh-FAP, further analysis showed the tumors produced by anti-BGN HGC27+CAFLCs/sh-control were evidently heavier and wider than those anti-BGN HGC27, anti-BGN HGC27+CAFLCs/sh-FAP, while two groups of anti-BGN HGC27, anti-BGN HGC27+CAFLCs/sh-FAP were not statistically significant in tumor weights and peritoneal dissemination.

Taken together, the results indicated that co-injection of anti-BGN HGC27+CAFLCs/sh-control rescued the inhibitory effect of knockdown BGN expression on subcutaneous tumor growth or intraperitoneal tumor dissemination, while co-injection of anti-BGN HGC27+CAFLCs/sh-FAP eliminated the rescued effect. Thus, GC cells-derived BGN and CAFLCs-derived FAP played a critical role in regulating tumor growth and intraperitoneal dissemination of GC in co-injection. IHC staining analysis and qRT-PCR were performed in different groups of intraperitoneal disseminated tumors. The result showed that anti-BGN HGC27 group inhibited FAP, BGN, and Ki67 expression of PM foci compared with anti-NC-BGN HGC27 group; while anti-BGN HGC27+CAFLCs/sh-control group promoted FAP, BGN, and Ki67 expression of PM foci compared with anti-BGN HGC27 group and anti-BGN HGC27+CAFLCs/sh-FAP group (Fig. 8c, 8e). Taken together, these results verified that GC cells-derived BGN promoted Ki67, FAP expression of intraperitoneal disseminated tumors in TME. At the same time, CAFLCs-derived FAP boosted the proliferation, BGN expression of GC cells to form a positive feedback loop, resulting in tumorigenesis and progression of GC PM in TME.

In this study, BGN was screened for further research using microarray sequencing. Both *in vitro* and *in vivo* experiments demonstrated that BGN promoted tumor progression and transformation of MCs into CAFLCs through the TLR2/TLR4/NF-κB signaling pathway. In turn, CAFLCs-derived FAP facilitated the proliferation, migration, invasion, and EMT of GC cells by activating JAK2/STAT3 signaling pathway. Activated STAT3 promoted the transcription of BGN in GC, thus forming a BGN/FAP-STAT3 positive feedback loop facilitating PM of GC (Fig. 8f).

## Discussion

According to Paget's “seed and soil” theory, metastasis occurs not only relies on competent cancer cells (the “seed”) but also requires a predetermined invitation by certain organs (the “soil”) 31. Thus, metastases are influenced by various factors and mysterious cellular crosstalk between cancer "seed" and "soil", now the "soil" has been known as the TME 32. The TME is rather complex, and extremely different from microenvironment of normal tissue, and HPMCs are a key component of the peritoneal TME for PM. The complex and dynamic bidirectional interactions between tumor cells and HMPCs-derived CAFs play an important role in PM. This study defined the molecular and cellular mechanisms that allow BGN to induce GC cells proliferation, migration, invasion, and EMT capacity, and convert HMrSV5 into CAFLCs. CAFLCs and CAFLCs-derived FAP were found, in turn, to facilitate the proliferation, migration, invasion, EMT, and BGN expression of GC cells through the JAK2/STAT3 signaling pathway. These results revealed that a positive feedback loop of cancer-TME-cancer promoted PM of GC through BGN/FAP-STAT3 interaction in TME.

Clinically, expression of BGN and FAP were evidently up-regulated in GC tissues compared with corresponding PANT, and BGN was positively correlated with FAP expression in GC tissues. IHC revealed that BGN and FAP in PM foci tissues were significantly higher than in primary GC lesion. BGN and FAP were also positively correlated with advanced TNM, poor tumor differentiation, LNM, and postoperative PM according to the 8th edition of the AJCC Cancer Staging Manual. In addition, BGN was defined as independent prognostic factors associated with poor clinical outcomes. Studies have found that high expression of BGN in colorectal cancer 33, GC 34, prostate cancer 35, lung cancer 36, endometrial cancer 37, and melanoma 38, and linked expression to poor prognosis. Moreover, high FAP expression is shown in GC 39, esophageal squamous cell carcinoma 40, colorectal cancer 41, pancreatic cancer 42, and non-small cell lung adenocarcinoma 43, and linked with poor prognosis. In accordance with Paget's “seed and soil” theory 31, high expression of BGN in GC tissues allows tumor cells to infiltrate and break through the stomach's serosa to colonize the peritoneum by transforming of HMPCs into CAFLCs through the interaction between cancer cells and stroma in the peritoneal TME.

PM derived from the gastrointestinal tract and ovary malignant tumors, various factors can transform HPMCs into CAFs, including malignant ascites 44, cancer-derived exosomes 4, 45, transforming growth factor-β 5, 46, peritoneal injury 13, tumor necrosis factor-α 47. In this study, BGN promoted the proliferation, migration, invasion, and EMT of GC cells. Recently studies showed that BGN enhanced migration of GC cells by the regulation of EMT 34. EMT enhances the tumor-initiating and potential of cancer cells metastasis 20. *In vitro* recovery experiment results from our study revealed that GC cells-derived BGN acted on the TLR2/TLR4 receptor of HMrSV5 and activated the downstream signaling molecule, NF-κB, which promoted the transformation of HMrSV5 into CAFLCs. Moreover, from the molecular mechanistic perspective, the Co-IP results demonstrated that BGN interacted with TLR2 and TLR4. Meanwhile, HMrSV5 after being co-cultured with Oe-BGN AGS increased p65 phosphorylation, facilitated p65 nuclear translocation to exert its biological functions. Hu et al. demonstrated that GC cells-secreted BGN could activate endothelial cells, stimulate tubular formation, and induce VEGF via TLR2/TLR4 and NF-κB signaling pathway 21. CAFs are a major constituent of TME with various functions, including ECM deposition and remodeling, crosstalk with tumor cells and TME, and progression of malignancies 9, 10, 28, 48. It was also demonstrated that HMrSV5-derived CAFLCs could boost the proliferation, migration, invasion, and EMT of GC cells, while HMrSV5 suppressed these effects.

Given the importance role of cytokines in cell-cell reciprocal interaction, FAP was identified as the most notably up-regulated cytokine in this study. Prior studies have found various concentration of secretory FAP in circulation 49. However, the plasma concentration of FAP in non-tumor patients was not significantly different in patients with a variety of cancers. This may be because FAP expression is transient in response to diverse stimuli, even though it is highly expressed in cancer tissues 30. In the current study, FAP concentrations in the supernatant of CAFLCs were significantly higher than HMrSV5. Our study demonstrated that CAFLCs and CAFLCs-derived FAP could boost the proliferation, migration, invasion, and EMT of GC cells. Liu et al. showed that exogenous FAP and HELF-derived FAP had a similar effect 39. Bioinformatics analysis of co-expression genes showed that interaction networks of BGN could interact with FAP 16. As expected, CAFLCs-derived FAP promoted BGN expression of GC cells in co-culture model. CAFs or CAFs-derived FAP are involved in the development of tumor-initiation and metastasis by complicated mechanisms in the TME, and JAK2/STAT3 signaling pathway plays an important role in this process 27, 29, 48, 50. In this study, CAFLCs-derived FAP promoted BGN expression and EMT of GC cells through JAK2/STAT3 signaling pathway. It is well known that STAT3 is a key transcription factor that regulates tumor progression and metastasis 51, 52. The findings shown here demonstrate an underlying association between FAP and BGN that is mediated by STAT3. CHIP and dual-luciferase reporter assays confirmed that STAT3 could directly bind two sites in the BGN promoter regions. In brief, this study provides strong evidence that FAP/STAT3-induced BGN activation is critical for GC cells proliferation, migration, invasion, and EMT capacity in this feedback loop, suggesting that the BGN/FAP-STAT3 signal axis plays a vital role in CAFLCs-medicated progression of GC PM.

In this study, BGN was screened for further research by microarray sequencing. *In vitro* experiments demonstrated that BGN promoted tumor progression and transformation of MCs into CAFLCs by TLR2/TLR4/NF-κB signaling pathway. In turn, CAFLCs-derived FAP facilitated the proliferation, migration, invasion, and EMT of GC cells by activating JAK2/STAT3 signaling pathway. Activated STAT3 could promote the transcription of BGN in GC, forming a BGN/FAP-STAT3 positive feedback loop that facilitated PM of GC. In conclusion, the mutual interaction between tumor cells and activated MCs mediated by a BGN/FAP-STAT3 positive feedback loop facilitates PM of GC, providing a poor prognostic factor which could contribute to peritoneal metastasis of gastric cancer.

## Supplementary Material

Supplementary table.

## Figures and Tables

**Table 1:** Correlation between BGN and FAP expression levels and clinicopathologic characteristics of GC patients.

Parameters	n (%)	BGN expression		*P*	FAP expression		*P*
		Low	High		Low	High	
Gender							
Male	39 (60.0)	8	31	0.347	13	26	0.583
Female	26 (40.0)	8	18		7	19	
Age (years)							
<60	22 (33.8)	6	16	0.722	5	17	0.315
≥60	43 (66.2)	10	33		15	28	
Tumor size (cm)							
<5	42 (64.6)	8	34	0.159	14	28	0.545
≥5	23 (35.4)	8	15		6	17	
Tumor differentiation							
Moderate/Well	23 (35.4)	9	14	**0.044**	12	11	**0.006**
Poor	42 (64.6)	7	35		8	34	
T stage							
T1-2	25 (38.5)	10	15	**0.023**	12	13	**0.017**
T3-4	40 (61.5)	6	34		8	32	
LNM							
N0-1	28 (43.1)	11	17	**0.017**	14	14	**0.003**
N2-3	37 (56.9)	5	32		6	31	
TNM stage^a^							
I/II	26 (40.0)	11	15	**0.007**	14	12	**0.001**
III/IV	39 (60.0)	5	34		6	33	
Intraoperative DM							
Absence	58 (89.2)	15	43	0.836	19	39	0.571
Presence	7 (10.8)	1	6		1	6	
CEA (ng/ml)							
<5	44 (67.7)	9	35	0.260	14	30	0.791
≥5	21 (32.3)	7	14		6	15	
CA19-9 (U/ml)							
<37	41 (63.1)	9	32	0.515	11	30	0.368
≥37	24 (36.9)	7	17		9	15	
Postoperative PM							
Absence	28 (43.1)	11	17	**0.017**	14	14	**0.003**
Presence	37 (56.9)	5	32		6	31	
Overall	65 (100)	16	49		20	45	

Notes: boldface indicates P < 0.05; ^a^, the 8th edition of the AJCC Cancer Staging Manual. Abbreviations: BGN, biglycan; FAP, fibroblast activation protein; T stage, tumor invasion stage; LNM, lymph node metastasis; TNM, tumor-node-metastasis; DM, distant metastasis; CEA, carcinoembryonic antigen; CA19-9, carbohydrate antigen 19-9; PM, peritoneal metastasis.

**Table 2:** Univariate and multivariate analyses of clinicopathologic parameters associated with progression-free survival and overall survival.

Parameters	Progression-free survival						Overall survival					
	Univariate analysis			Multivariate analysis			Univariate analysis			Multivariate analysis		
	HR	95% CI	*P*	HR	95% CI	*P*	HR	95% CI	*P*	HR	95% CI	*P*
Gender (Female vs Male)	1.029	0.599-1.768	0.918				0.958	0.558-1.646	0.877			
Age (<60 vs ≥60)	1.005	0.568-1.780	0.985				0.965	0.573-1.791	0.965			
Tumor size (≥5 cm vs <5 cm)	1.011	0.582-1.758	0.969				1.001	0.576-1.740	0.997			
Tumor differentiation (Poor vs Moderate/Well)	1.030	0.585-1.813	0.919				0.948	0.579-1.793	0.948			
T stage (T3-4 vs T1-2)	1.869	1.048-3.335	**0.034**	0.668	0.289-1.542	0.344	1.834	1.033-3.256	**0.038**	0.675	0.287-1.589	0.368
LNM (N2-3 vs N0-1)	1.609	0.936-2.766	0.085				1.580	0.920-2.714	0.097			
TNM stage^a^ (III/IV vs I/II)	2.226	1.262-3.925	**0.006**	1.129	0.480-2.654	0.781	2.201	1.250-3.877	**0.006**	1.061	0.441-2.554	0.894
Intraoperative DM (Presence vs Absence)	6.452	2.588-16.083	**0.000**	6.400	2.251-18.198	**0.000**	6.269	2.525-15.562	**0.000**	7.583	2.524-22.782	**0.000**
CEA (High vs Low)	0.800	0.442-1.446	0.460				0.814	0.450-1.472	0.496			
CA19-9 (High vs Low)	1.228	0.707-2.131	0.466				1.249	0.719-2.170	0.429			
Postoperative PM (Presence vs Absence)	6.873	3.530-13.384	**0.000**	6.248	2.998-13.020	**0.000**	6.494	3.391-12.434	**0.000**	5.850	2.817-12.146	**0.000**
BGN (High vs Low)	4.095	1.958-8.565	**0.000**	4.733	2.153-10.408	**0.000**	3.910	1.877-8.145	**0.000**	4.851	2.156-10.914	**0.000**
FAP (High vs Low)	2.459	1.318-4.591	**0.005**	1.809	0.899-3.640	**0.096**	2.497	1.328-4.696	**0.005**	2.018	0.988-4.120	**0.054**

Notes: boldface indicates *P* < 0.05; ^a^, the 8th edition of the AJCC Cancer Staging Manual. Abbreviations: BGN, biglycan; FAP, fibroblast activation protein; T stage, tumor invasion stage; LNM, lymph node metastasis; TNM, tumor-node-metastasis; DM, distant metastasis; CEA, carcinoembryonic antigen; CA19-9, carbohydrate antigen 19-9; PM, peritoneal metastasis.

## Abbreviations

CAFs: cancer-associated fibroblasts
CAFLCs: cancer-associated fibroblasts like cells
GC: gastric cancer
PM: peritoneal metastasis
EMT: epithelial-mesenchymal transition
MCs: mesothelial cells
BGN: biglycan
FAP: fibroblast activation protein
HPMCs: human peritoneal mesothelial cells
MMT: mesothelial-to-mesenchymal transition
α-SMA: α-smooth muscle actin
ECM: extracellular protein
TME: tumor microenvironment
TLR: Toll-like receptor
PANT: paired adjacent normal tissues
rFAP: recombinant human FAP protein
IHC: Immunohistochemistry
anti-BGN: lentivirus mediated knockdown of BGN vectors
anti-NC-BGN: lentivirus mediated negative control knockdown of BGN vectors
Oe-BGN: lentivirus mediated overexpression of BGN vectors
Oe-NC-BGN: lentivirus mediated negative control overexpression of BGN vectors
GFP: green fluorescent protein
sh: short hairpin knockdown RNA
qRT-PCR: quantitative real-time polymerase chain reaction
GAPDH: Glyceraldehyde-3-phosphate dehydrogenase
WT: wild type
MUT: mutation
CHIP: chromatin immunoprecipitation
IF: immunofluorescence staining
CCK8: Cell Counting Kit-8
OS: overall survival
PFS: progression-free survival
T stage: tumor invasion stage
LNM: lymph node metastasis
TNM: tumor-node-metastasis
DM: distant metastasis
CEA: carcinoembryonic antigen
CA19-9: carbohydrate antigen 19-9
h: hours
DMEM: Dulbecco's modified Eagle's medium
FBS: fetal bovine serum
DMSO: Dimethyl sulfoxide
ELISA: enzyme-linked immunosorbent assay
PBS: phosphate-buffered saline
TBST: tris-buffered saline tween-20
SEM: standard error of the mean
SPSS: Statistical Product and Service Solutions
NC: negative control
Co-IP: Coimmunoprecipitation
STAT3-Ex: human plasmid expression STAT3
STAT3-NC: human plasmid negative control expression STAT3
IgG: Immunoglobulin G
