# Supplementary material for: BGN/FAP/STAT3 positive feedback loop mediated mutual interaction between tumor cells and mesothelial cells contributes to peritoneal metastasis of gastric cancer
Source: Int J Biol Sci. 2023 Jan 1;19(2):465–83. doi: 10.7150/ijbs.72218 (PMC9830517; doi:10.7150/ijbs.72218)
Supplement: Supplementary file 1 — Supplementary table. [file ijbsv19p0465s1.pdf]

Table S1: The sequence of the primers for quantitative RT-PCR

Gene	Primer Sequence (5' – 3')
BGN	F: AGGAGGCGGTCCATAAGAAT
	R: AGGGTTGAAAGGCTGGAAAT
FAP	F: TGAACGAGTATGTTTGCAGTGG
	R: GGTCTTTGGACAATCCCATGT
TGF-β1	F: CCATCCCGCCCACTTTCTAC
	R: AGCTCAATCCGTTGTTCAGGC
EGF	F: TCTGAATGTCCCCTGTCCCACG
	R: CTGCGACTCCTCACATCTCTGC
IL-8	F: AAACCACCGGAAGGAACCAT
	R: CCTTCACACAGAGCTGCAGAAA
IL-6	F: AATAACCACCCCTGACCCAAC
	R: ACATTTGCCGAAGAGCCCT
HGF	F: GCTATCGGGGTAAAGACCTACA
	R: CGTAGCGTACCTCTGGATTGC
CHIP primer site 1	F: CTCTACCGACATATTTCTCCACG
	R: ACCACATATGCCCTTTCCAG
CHIP primer site 2	F: GACTCGCTGGATCCTTCGTC
	R: ACTTCATGTAGGCTCCTGGC
CHIP primer site 3	F: GCCTACATGAAGTCCTTCCAGA
	R: GCCTAAGCAAGGAGGTGGTT
GAPDH	F: CTGTTCGACAGTCAGCCGCATC
	R: GCGCCCAATACGACCAAATCCG
